# The relative frequency of odontogenic tumors: A study of 376 cases in a Brazilian population

**DOI:** 10.4317/medoral.21285

**Published:** 2017-02-04

**Authors:** Rafael Lima-Verde-Osterne, Eveline Turatti, Renata Cordeiro-Teixeira, Roberta Barroso-Cavalcante

**Affiliations:** 1 DDS, MSc, Auxiliar Professor. Department of Pathology, School of Medicine, University of Fortaleza, CE, Brazil; 2 DDS, PhD, Titular Professor. Department of Oral Pathology, School of Dentistry, University of Fortaleza, CE, Brazil; 3 DDS, PhD, Assistant Professor. Division of Oral Radiology, School of Dentistry, University of Fortaleza, CE, Brazil; 4 DDS, PhD, Assistant Professor. Division of Oral Pathology, School of Dentistry, University of Fortaleza, CE, Brazil

## Abstract

**Background:**

Odontogenic tumors (OTs) are rare lesions, exclusive of the jaws, that are derived from epithelial and/or ectomesenchymal elements of the tooth-forming apparatus. Their biological behavior is heterogeneous, including hamartomatous tissue proliferation, benign nonaggressive and aggressive neoplasms, and malignant tumors with metastatic capacity. The aim of this study was to describe the relative frequency of odontogenic tumors in a Brazilian population. In addition, a review of the literature identified studies on odontogenic tumors that follow the 2005 World Health Organization.

**Material and Methods:**

A total of 376 cases of odontogenic tumors from an oral pathology service were reviewed about age, gender, anatomic site and histologic diagnosis.

**Results:**

Keratocystic odontogenic tumors (31.6%) were the most common, followed by ameloblastoma (28.5%), and odontoma (22.6%). The mean age was 32.2 years, and more than half the patients (52.1%) were in the second and third decades of life. The male to female ratio was 1:1.37, with a maxilla to mandible ratio of 1:2.08.

**Conclusions:**

The variation in relative frequency of tumors observed among the several series, including the present study, is probably due in part to cultural differences between geographic areas but also to the study design.

**Key words:**Pathology, epidemiology, odontogenic tumors.

## Introduction

Odontogenic tumors (OTs) are rare lesions, exclusive of the jaws, that are derived from epithelial and/or ectomesenchymal elements of the tooth-forming apparatus. Their biological behavior is heterogeneous, including hamartomatous tissue proliferation, benign nonaggressive and aggressive neoplasms, and malignant tumors with metastatic capacity (1).

Since 2005, epidemiological OT studies have followed two major classificatory systems. The majority of these studies are based on the 2005 WHO classification of tumors (1-13). On the other hand, other studies still followed the 1992 WHO classification (14-19). The changes in the 2005 classification included terminology, classification as benign or malignant or assignment to relevant subgroups, in particular the benign tumors (20). However, the main difference for relative frequencies studies was the addition of the odontogenic keratocyst to the benign OTs, termed as keratocystic odontogenic tumor (KCOT). This redefinition produced a huge increase in the frequency and prevalence of OTs (12), without, however, impacts on the treatment conventions for the KCOTs (20).

Knowledge of prevalence of the OTs can be extremely valuable both for pathologists and clinicians when developing differential diagnosis (4). Reports on the relative frequency of OTs from different countries show a distinct geographic variation (6,8,12,13). Although some reports have been published concerning the relative frequency of OTs in Brazil (3,8,10,12,21,22), few studies are based on large samples from a single institution (12,22). Thus, the objective of this study is to describe the relative frequency of OTs at the Oral Pathology Laboratory at the University of Fortaleza (Ceará State, Brazil) over a period of 12 years, based on the 2005 WHO classification (1). We then compare these results with previous studies published from other parts of the world, including one previous study from the same state of Ceará in Brazil.

## Material and Methods

This cross-sectional study was approved by the institutional Ethics Commitee (ethics approval number 1104619). A total of 9100 biopsy records were reviewed and 376 (4.1%) met the inclusion criteria for 2005 WHO classification for OTs (1). Recurrent tumors as well as cases with repeated biopsies of the same lesion were excluded.

Within this laboratory, all diagnosis requires the consensus of two pathologists. The data collected from the clinical records and histopathological report included a unique biopsy number, patient age at the date of the biopsy, gender, tumor site and histopathological diagnosis. Personal or identifiable information was not recorded to maintain anonymity.

Age at the time of the diagnosis was treated as a continuous variable, but also categorized into eight age groups, with a 10-year interval. All tumor sites were classified into anterior maxilla, posterior maxilla, anterior mandible, or posterior mandible. The histopathological diagnoses were adapted to the 2005 WHO classification of OTs (1). The resulting data set was analyzed using the Statistical Package for Social Science, version 20.0 (SPSS, Chicago, IL).

Comparison studies were identified from the existing literature (PubMed Database) of series of odontogenic tumors published between 2005 and 2015. These studies all used the 2005 WHO classification (1), and those that did not mention the malignant tumors or failed to include any of the most prevalent benign tumors were excluded.

## Results

In the data set, 99.2% of the tumors were benign and 0.8% were malignant. Primary intraosseous squamous cell carcinoma (PIOSCC) (0.5%) and ameloblastic carcinoma (AC) (0.3%) were the only two malignant entities. Keratocyst odontogenic tumor (KCOT) was the most frequent benign tumor (31.6%) followed by ameloblastoma (28.5%), odontoma (22.6%), and odontogenic myxoma (4.5%). Other benign OTs comprised between 0.3% and 4.3%. Regarding tumor histogenesis, 65.9% of the tumors were classified as OTs of odontogenic epithelium with mature fibrous stroma without odontogenic ectomesenchyme; 27.9% were OTs of odontogenic epithelium with odontogenic ectomesenchyme, with or without hard tissue formation; and 6.1% were OTs of mesenchyme and/or odontogenic ectomesenchyme with or without odontogenic epithelium.

 Table 1 shows the relative frequency and gender distribution of OTs. In three biopsies (0.8%), the gender of the patient was not provided and thus not included in the results. There were 157 (41.8%) male specimens and 216 (57.4%) female specimens, for a gender ratio of 1:1.37. For the three most frequently observed tumors (KCOT, ameloblastoma, and odontomas), the male:female ratios are 1:1.05, 1:1.3, and 1:1.89) respectively.

**Table 1:**
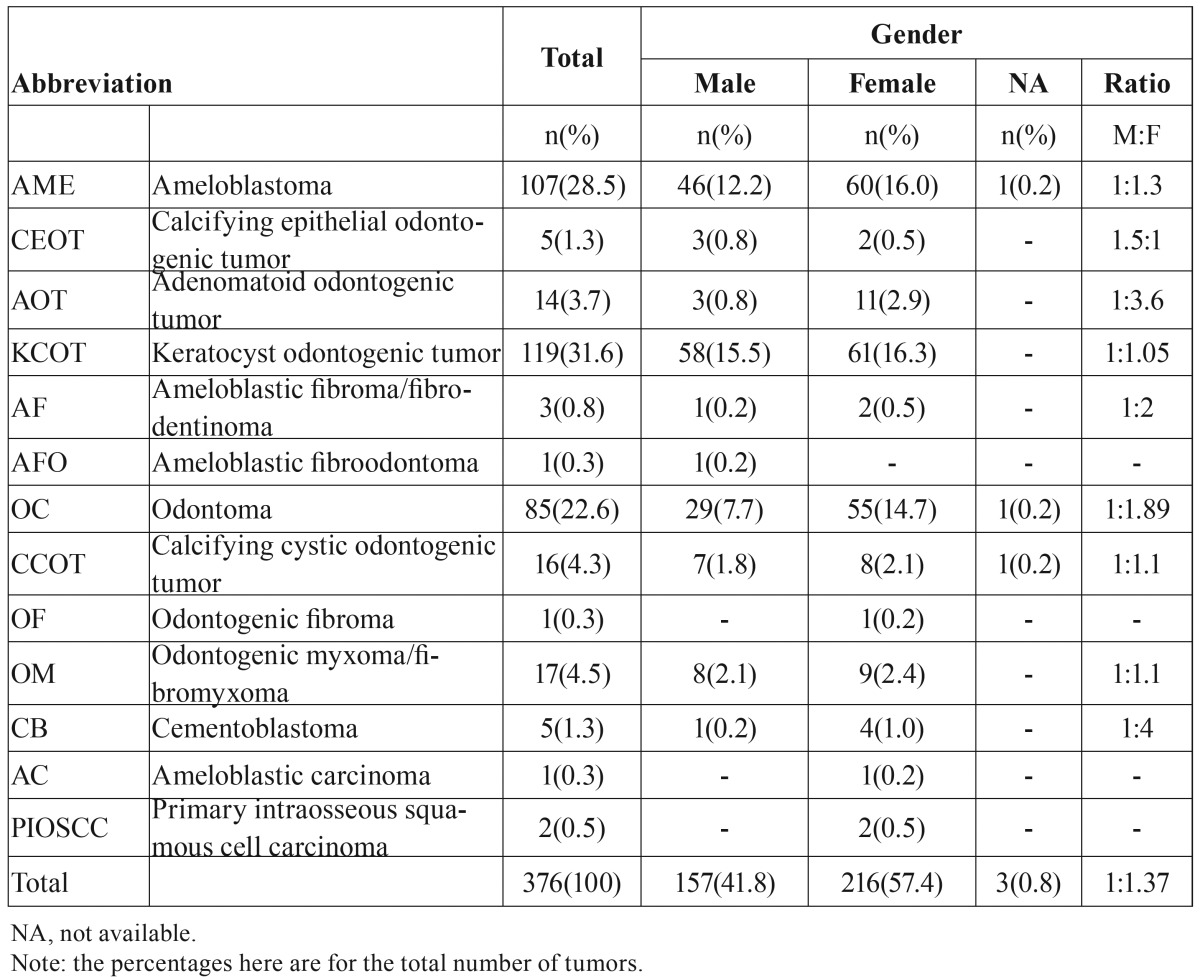
Gender distribution of odontogenic tumors.

As shown in  Table 2, the age of patients at diagnosis ranged from 3 to 99 years with a mean of 32.2(±18.7) years. The peak age categories of occurrence are in the second and third decades of life, which comprised more than half the diagnosed OTs biopsies (52.1%). Overall, the KCOT and ameloblastoma show the higher relative frequencies in all decades except the first and second, where odontoma occurred with greater frequency. The third decade is the only one where the ameloblastoma is more common than the KCOT.

**Table 2:**
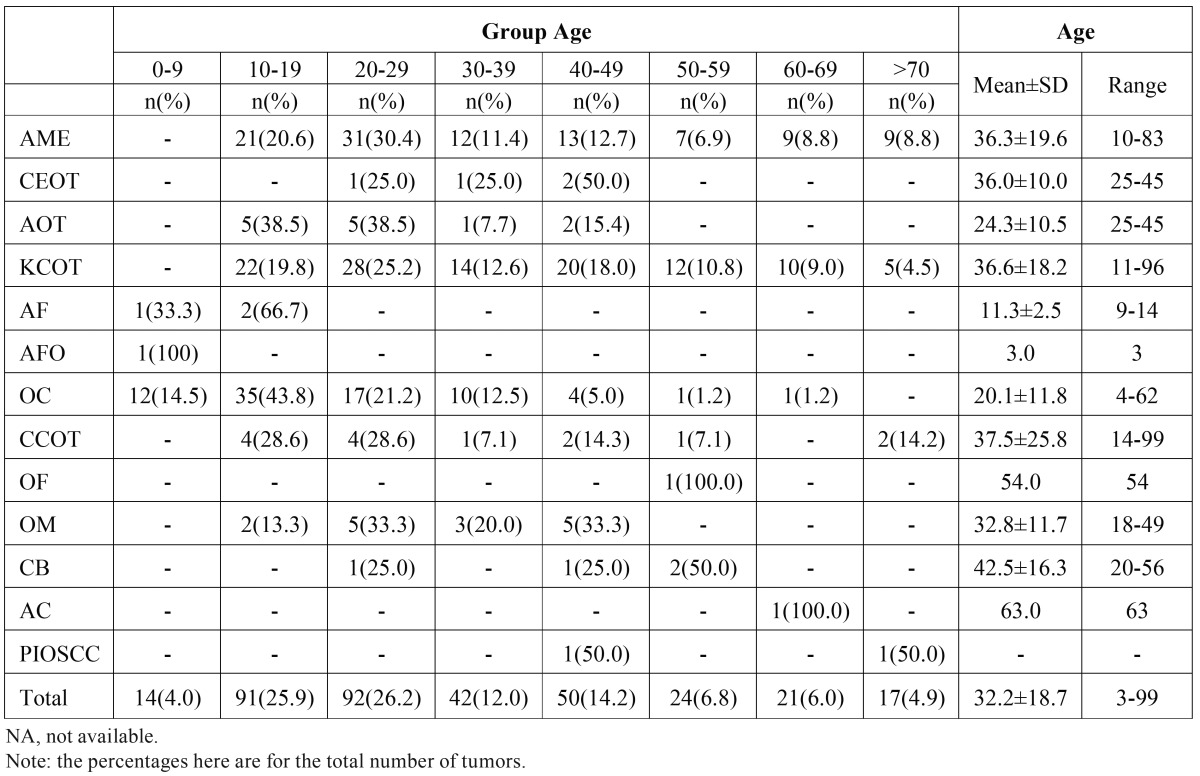
Age distribution of odontogenic tumors.

 Table 3 shows the site distribution of each type of OT, where 248 (65.9%) tumors were located in the mandible and 119 (31.70%) tumors in maxilla, for an overall maxilla-mandible ratio of 1:2.08. In maxilla, the anterior region was the predominant site of involvement, mostly contributed by odontoma. The posterior region of the mandible was the frequent site of involvement in 190 (50.5%) tumors. Ameloblastoma showed a high mandibular predilection, whereas AOT was more common in the anterior region of the maxilla.

**Table 3:**
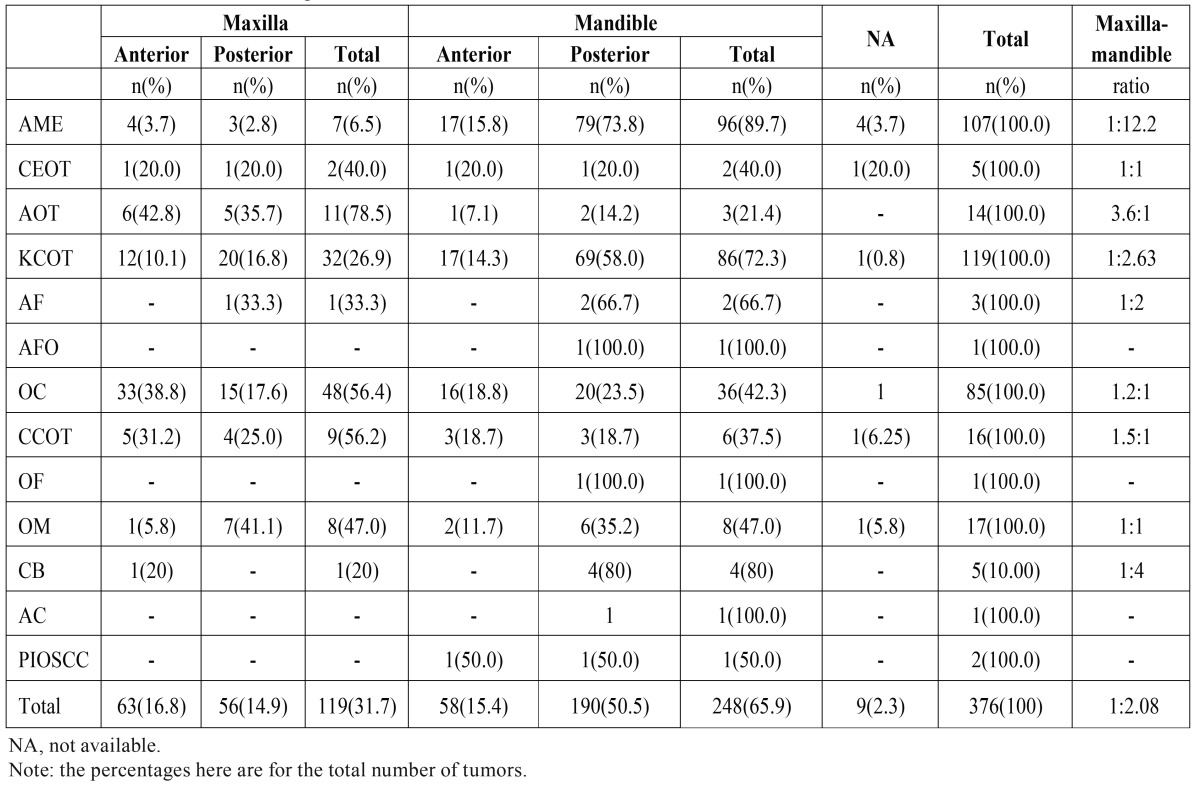
Site distribution of odontogenic tumors.

## Discussion

This study describes a series of OTs from the Oral Pathology Laboratory of the University of Fortaleza, which is the major referral center for biopsied lesions in Ceará, Northeast of Brazil, a state with more than 8.8 million inhabitants. Our sample is the largest yet analyzed in Brazil. There is, however, a previous study from the population of Ceará conducted by Osterne *et al.* (8) albeit using a smaller sample.

Despite the fact that some studies published after 2005 did not observe the latest WHO classification for OTs, (14-18) the majority of studies have employed the 2005 classification (2-13). Servato *et al.* (12) showed that the average proportion of OTs among oral and maxillofacial lesions evaluated by histopathology increased from 3% (±2.9%) in studies that used the 1992 WHO (19) classification of tumors to 4.0% (±1.3%) in those with the later classification. This is in concordance with the present study, where the relative frequency of OTs is 4.1% of the total biopsied specimens between January 2001 and December 2013. However, there seems to be significant geographical variation in the frequency of OTs, with lower rates in Europe (15) (0.84%) and North America (14) (1.2%) and higher rates in Asia (18) (4.1%) and Africa (23) (9.3%). In Brazil, the frequency rates range between 1.3% and 4.76%, most likely due to inherent regional disparities between the Northeast and the Center-South (3,10,12).

Across all the studies reviewed for comparison, the most frequent OT is ameloblastoma (39.1%), followed by KCOT (32.1%), and odontoma (10.2%) ( Table 4). These rates differ from the order of magnitude in our study where KCOT is more frequent (31.6%), followed by ameloblastoma (28.5%) and odontoma (22.6%). These differences in the rankings of frequencies, especially for KCOT and ameloblastoma, appear to follow a distinct geographical pattern. In the studies conducted in the Western Hemisphere (including Brazil) KCOT is reported with the greatest frequency, while in the Asian and African studies, ameloblastoma is the most frequent OT (2,3,5-7,9-12).

**Table 4:**
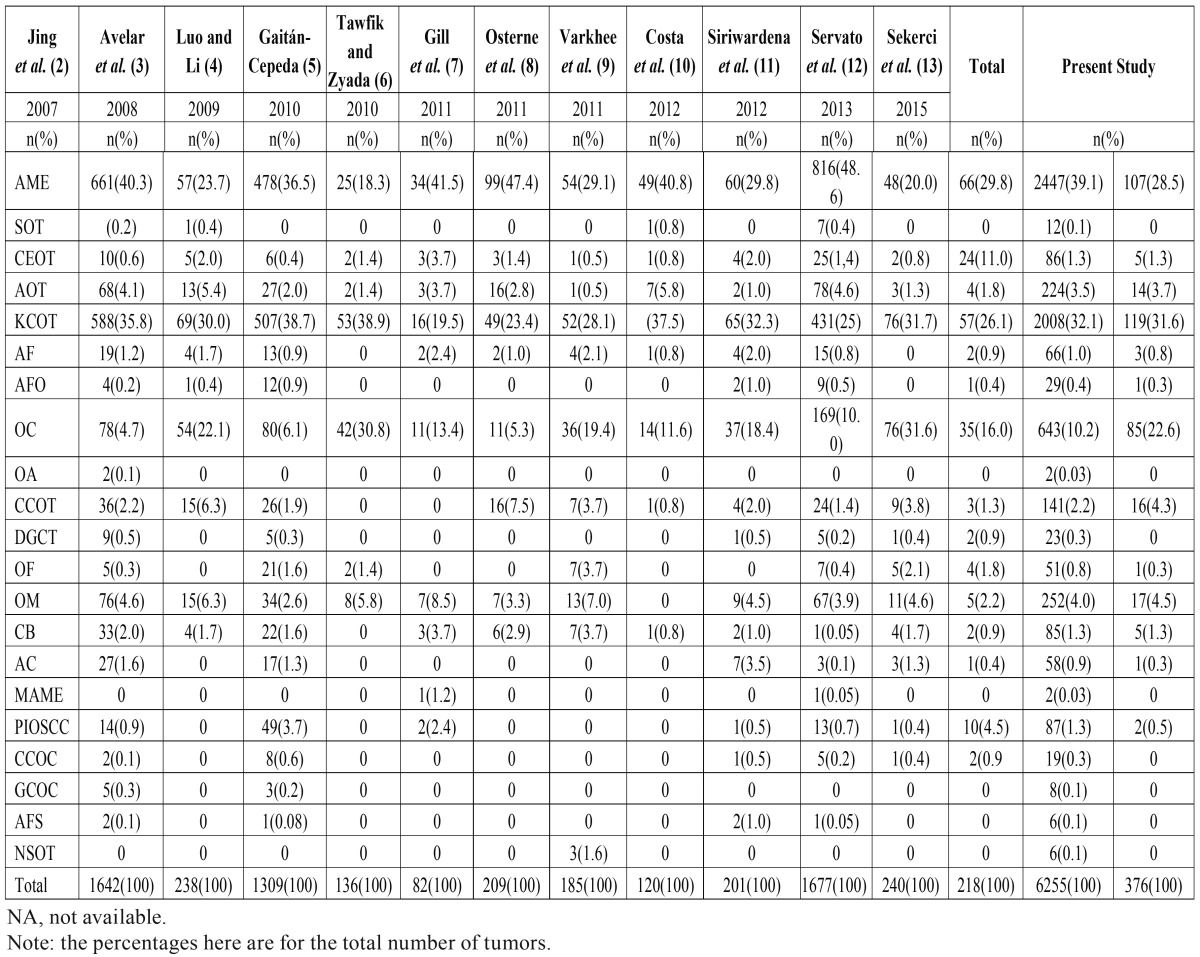
Distribution of odontogenic tumors by diagnosis.

Odontoma is the third most common OT, although the frequencies differ meaningfully between studies. Fregnani *et al.* (24) argue that differences in results are due to data sources, rather than geography. Medical hospitals underestimate odontoma frequencies and overestimate the rates of tumors that require extensive surgical procedures. Moreover, in several developing countries, odontomas are occasionally not registered or sent for histological confirmation. In the current study, the relative frequency for odontomas was 22.6% and this is in concordance with Brazilian series conducted by Avelar *et al.* (3) (22.1%), Costa *et al.* (10) (18.4%), and Osterne *et al.* (8) (19.4%).

In general, the comparison of studies found wide variations in the occurrence of the less frequent tumors, such as odontogenic myxoma (2.2% - 6.3%), AOT (0.5% - 5.8%), and cementoblastoma (0% - 3.7%). Despite some divergence, our results are consistent with the averages of the other studies reviewed here (4.5% vs 4.0% respectively for odontogenic myxoma; 3.7% vs 3.5% for AOT; and 1.3% vs 1.3% for cementoblastoma). It should be mentioned that our relative frequency for calcifying cystic odontogenic tumor (4.3%) were slightly higher compared to other studies (2.2%), however, these differences are confirmed in other Brazilian studies-Osterne *et al.* (8), Servato *et al.* (12), and Avelar *et al.* (3), with rates of 3.7%, 3.8% and 6.3%, respectively. Also, the low occurrence of calcifying epithelial odontogenic tumor (1.3%), ameloblastic fibroma (0.8%), ameloblastic fibro-odontoma (0,3%), and odontogenic fibroma (0.3%) was comparable to findings reported elsewhere (7,11).

Malignant OTs in the present study represented 0.8% of the total OTs. This relative frequency is similar to other reports from the Western Hemisphere countries that ranged from 0% to 1.1% (25-27), but contrast with the significantly higher rates from Africa (23) and China (4), 5.8% to 5.9% respectively.

Regarding gender, the literature states that male patients are more affected by OTs than females ( Table 5). However, it seems that the gender distribution of OTs also shows a geographic variation, as reported in studies from South America (3,5,8,12,27), including the present study, where higher female rates were identified.

**Table 5:**
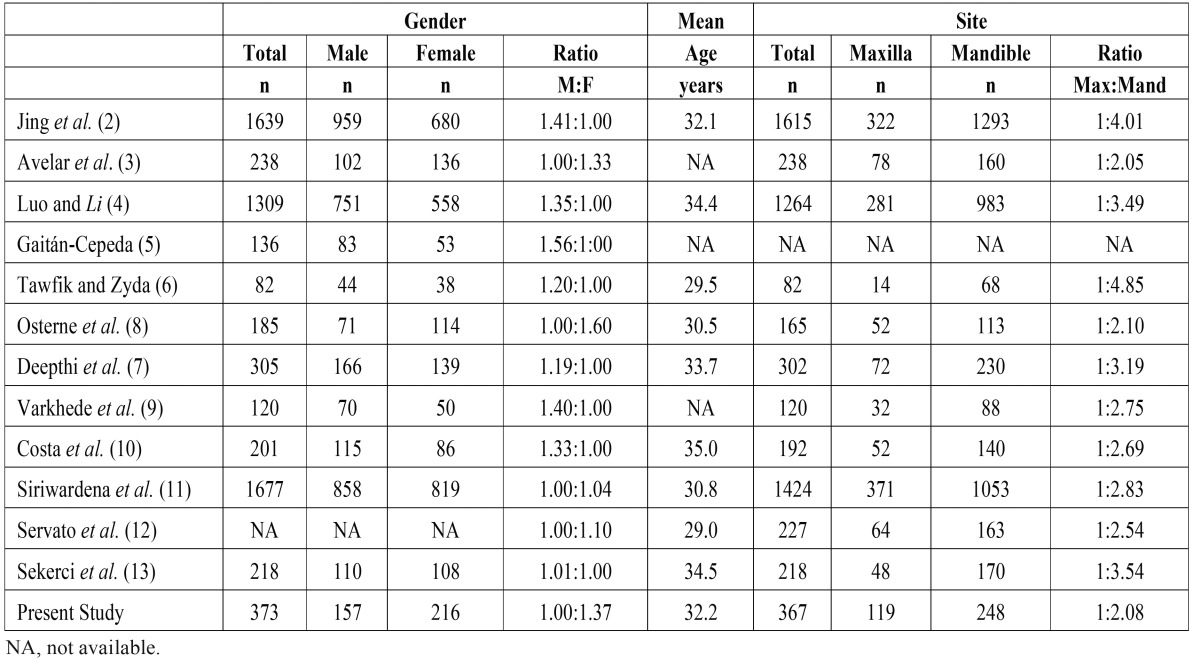
Gender, mean age, and site distribution of odontogenic tumors.

Comparison rates across studies suggest that frequency rates do not vary in terms of average age, but there are strong geographic differences with regard to the age decade in which the different OTs occur. The Brazilian studies show that the second decade of life is the most affected (3,8,12), followed by the third decade. In Egypt, India and Sri-Lanka, on the other hand, the order of age decades is reversed and the third decade shows the highest rates (6,9,11). In the present study, the second and the third decades of life were the most common and equally affected. In China and Turkey (2,4,13), the third and fourth decades were most afflicted. These variations clearly mirror the percentages of KCOT, ameloblastoma and odontoma in each study. Because odontoma occurs in younger patients, in studies where the relative frequency of this tumor is high, the second and third decade of life are the most affected (3,8,9,12). Where high frequencies of ameloblastoma are present, the age occurrence shifts to the third and fourth decades (2,4,13). In contrast, KCOT is distributed more uniformly across age.

Specifically with regard to ameloblastoma, the mean age of occurrence does show significant variation across countries. Reichart *et al.* (28) report a mean age in developing countries to be 27.7 years and in developed countries to be 39.1 years. In the current study, the mean age of occurrence for ameloblastoma (36.6 years) was closer to that reported by Reichart *et al.* (28) for developed countries, showing that there might be others factors involved. It has been previously show by Ledesma-Montes *et al.* (29), in a study of 163 amelobastoma cases from Latin-America, that solid ameloblastoma has a higher mean age of occurrence (41.4 years) than unicystic ameloblastoma (26.3 years), in fact, not a single case of solid ameloblastoma was found in patient younger than 20-years old in their series. In the present study, information about the subtypes of ameloblastomas included is not available, and this may by a limitation factor for explaining the possible differences between the mean age of this and other series. Other studies of ameloblastoma from different regions, with homogeneous and actually accepted criteria to differentiate unicystic ameloblastoma and solid ameloblastoma could help to clarify if there are geographics or ethnics differences in occurrence of ameloblastomas.

Odontoma is frequently found in the first and second decade of life with the mean age of 18.4 years (14). The slightly higher mean age (20.1 years) seen in the present study for odontomas probably reveals the fact that Brazilian patients do not frequently undergo routine panoramic X-ray and so odontomas are not diagnosed as early. A systematic review of KCOT revealed a mean age of 36.5 years with a peak of incidence in the second and third decades of life (30). Our mean age were similar (36.6 years), but with a smoother distribution over the decades. The other OT that shows a marked pattern by an age group is AOT (31), where 65% of these tumors occur in the second decade of life. In our data, 77% of AOTs were equally distributed between the second and the third decades of life. That delay in the age of diagnosis was probably due to the fact that, likewise odontomas, AOT exhibits self-limiting growth and does not require urgent treatment.

Most OTs were found in the posterior region of the mandible. The current study presented a maxilla:mandible ratio of 1:2.08. A higher maxilla:mandible ratio (1:3.6) were reported in literature (8) which reflects the relatively higher rates of ameloblastomas in large samples (2,4,11). Ameloblastomas, KCOT and cementoblastomas are widely known to affect more the posterior region of mandible (1,8,14). In accordance with literature, our study shows that 73.8% of the ameloblastomas and 57.9% KCOTs occur in that region. Cementoblastomas, also showed a strong mandible occurrence (80%), despite their relative scarcity in the sample as a whole. In contrast, AOT and odontomas occur more frequently in the maxilla, accounting for 78.5% and 56.4% of the sites, respectively. These observations are confirmed by Gupta & Ponniah *et al.*(18), Avelar *et al.* (3), Deepthi *et al.* (7), but not by Buchner *et al.* (14) and Jing *et al.* (2).

While most studies show the predominant occurrence of odontogenic myxomas in the mandible (8), our results reveal an equal distribution of odontogenic myxoma between maxilla (47.0%) and mandible (47.0%). No conclusions could be drawn with regard to gender, age and anatomic site predilection for the rarest OTs (CEOT, AF, OF, CB, AC, and PIOSCC) owing to paucity of cases.

It was interesting to see that our results are in accordance with a previous study conducted by Osterne *et al.* (8) in the same region – state of Ceará/Brazil - with data from 2001 to 2005. The relative frequencies of the most common tumors were similar, and the maxila:mandible ratio was almost identical (1:2.08 vs 1:2.1). In both studies, the second and the third decades of life were the most affected, which taken together accounted for 52.1% and 51.27% of the total number of tumors between our study and that of Osterne *et al.* (8). The gender distribution showed the larger difference with a male:female ratio of 1:1.37 for our study compared to 1:1.6 for Osterne *et al.* (8).

Lastly, it is important to highlight that although we serve the majority of the State of Ceará, there are some biopsies analyzed in other laboratories. Also, because the size and age of patient population are unknown, we cannot calculate prevalence and incidence rates. Nevertheless, this is the largest study of its kind in the country, so it provides valuable information on the types of tumors and percentages of each type of tumor by age, gender, and anatomic site.

In conclusion, overall our results are shared by the others Brazilian studies but show marked geographic variation to those reported from Asia and Africa. The variations in relative frequency of tumors observed among the several series, including the present study, are probably due in part to underlying cultural differences between geographic areas but also to differing elements of the study design, which would require further research to determine.
